# Pharmacokinetics and pharmacodynamics integration of danofloxacin against *Eschrichia coli* in piglet ileum ultrafiltration probe model

**DOI:** 10.1038/s41598-020-80272-7

**Published:** 2021-01-12

**Authors:** Yuqi Yang, Ping Cheng, Tianshi Xiao, Jargalsaikhan Ulziikhutag, Hongxiao Yu, Jiarui Li, Ruimeng Liu, Ishfaq Muhammad, Xiuying Zhang

**Affiliations:** 1 Pharmacology Teaching and Research Department, School of Basic Medicine, Guizhou University of Traditional Chinese Medicine, University Town, Dongqing Road, Huaxi District, Guiyang, People’s Republic of China; 2 Heilongjiang Key Laboratory for Animal Disease Control and Pharmaceutical Development, Faculty of Basic Veterinary Science, College of Veterinary Medicine, Northeast Agricultural University, 600 Changjiang Road, Xiangfang District, Harbin, 150030 Heilongjiang People’s Republic of China

**Keywords:** Microbiology, Antimicrobials

## Abstract

Improper use of antibiotics results in poor treatment and severe bacterial resistance. In this study, ultrafiltration probes were successfully placed in the ileum of piglets with the aid of anesthetic. After the fluoroquinolone antimicrobial drug danofloxacin (DAN) was intramuscularly administered, blood and ileum ultrafiltrate were collected at different time points and then determined by High Performance Liquid Chromatography (HPLC). Pharmacokinetics (PK) parameters for plasma and ileum ultrafiltrate were calculated by WinNonlin software. The DAN concentration in ileum ultrafiltrate was much higher than that in plasma during the period 1.2–48 h. The DAN concentration in plasma reached its maximum at 1.10 ± 0.03 h, but reached at 6.00 ± 0.00 h in the ileum ultrafiltrate. The mean C_max_ of the ileum is 13.59 times that of plasma. The elimination half-life (T_1/2β_) in the ileum ultrafiltrate (6.84 ± 1.49 h) was shorter than those in plasma (7.58 ± 3.20 h). The MIC, MBC and MPC of DAN in MH broth against *Escherichia coli* (O_158_) were 0.5 µg/mL, 0.5 µg/mL and 4 µg/mL, respectively. Both in vitro and ex vivo kill curves indicated that the killing mechanism of DAN against *E. coli* is concentration-dependent. The AUC/MPC ratio is 21.33 ± 2.14. Mean PK/PD index (AUC_24h_/MIC) for ileum ultrafiltrate that achieved bacteriostatic, bactericidal, and eradication were 99.85, 155.57, and 218.02 h, respectively. Three different dosages (1.49 mg/kg, 2.42 mg/kg, and 3.24 mg/kg) were calculated respectively based on AUC_24h_/MIC ratio above, which might provide a novel approach to the rational design of dosage schedules.

## Introduction

*Escherichia coli* (*E. coli*), a prominent commensal bacterium in gastrointestinal tract of animals and humans, acts not only as an important human pathogen associated with intestinal diseases but also a causative agent resulting in diarrhoea in animals^1,2^. Danofloxacin (DAN) is a fluoroquinolone active against a broad spectrum of bacteria^3^.

The MIC is the lowest concentration of antimicrobial agent that inhibits growth of the organism in the microdilution wells as detected by the unaided eye; the minimal bactericidal concentration (MBC) is the minimal concentration of drug needed to kill most (≥ 99.9%) of the viable organisms after incubation for a fixed length of time (generally 24 h); the mutant prevention concentration (MPC) is defined as the concentration preventing growth at a high (˃ 10^9^ CFU/mL) inoculum using agar dilution methodology^4^.

Antibiotic resistance has become a global health threat for decades. It has been upgraded by major world health organizations to one of the top health concerns of the twenty-first century^5^. In the prevention and control of drug resistance, the most important is to develop new antibiotics and to design effective dosage schedules. It is regrettable that the speed of antibacterial drug development is far behind the speed of drug resistance. From 1985 to 2016, the number of antibiotic resistant bacteria infections is increasing whereas the development of new antibiotics is constantly decreasing^6^. Moreover, the development of drug resistance is unimaginable, and drug resistant bacteria appear in the early stage of clinical application of many new drugs^7^. Therefore, a rational dosage schedule must be designed to reduce the emergence of drug resistance. The design of rational dosage schedule is dependent on (i) the linkage of Pharmacokinetics (PK) data to ex vivo or in vivo pharmacodynamic (PD) data generated in animal models or in clinical trials and (ii) The killing mechanism of the drug against bacteria. The integrated PK-PD parameters the area under curve (AUC_24h_)/MIC and maximum concentration of drug (C_max_)/MIC are therefore closely related to a successful treatment outcome^3^.

However, few previous publications have described the PK of DAN in animal model, especially in piglets. The objectives of this study were (1) to implant an ultrafiltration probe into the ileum of pig to continuously collect intestinal fluid; (2) to establish plasma and ileum PK data for DAN in piglet after intramuscular (i.m.) treatment; (3) to determine integrated PK-PD parameters (AUC_24h_/MIC and C_max_/MIC) for DAN in vivo; (4) to establish the AUC/MIC ex vivo that produce bacteriostatic, bactericidal, and eradication by using the inhibitory effect sigmoid E_max_ equation; and (5) to calculate respectively different dosages based on AUC_24h_/MIC ratio that might provide a novel approach to the rational design of dosage schedules which provide maximal efficacy and minimizes opportunity for the emergence of resistant strains.

## Materials and methods

### Ethical statement

All of the experiments were conducted under the supervision of the Harbin Veterinary Research Institute of the Chinese Academy of Agricultural Sciences in accordance with animal ethics guidelines and approved protocols. The Harbin Veterinary Research Institute Animal Ethics Committee approval number was SYXK (Hei) 2012-2067.

### Pathogenicity test

The laboratory preserved strains (HP65, HP189, HP232 and HP501) were conveniently selected for pathogenicity test. Pathogenicity test was performed with 39 Kunming mice (weighted 32 ± 3 g, specific pathogen free grade) purchased from Experimental Animal Centre of Harbin Medical University. Every three mice were intraperitoneal administrated with 200µL *E. coli* suspension (10^7^, 10^8^, and 10^9^ CFU/mL). The blank control group (n = 3) were intraperitoneal administrated with 200 µL sterile broth. The mice were observed every 3 h after the injection up to 72 h. If there were three mice dead in a group, the inoculant given to this group was considered to be of high pathogenicity.

### Bacterial strain, antimicrobials, and chemicals

According to the results of pathogenicity test, an EPEC *E. coli* strain HP501 (O_158_) was selected for subsequent PD experiments. The reference strain *E. coli* ATCC25922 strain was purchased from the NATIONAL CENTER FOR MEDICAL CULTURE COLLECTIONS (Beijing, China). Mueller Hinton broth was purchased from Qingdao Hope Bio-Technology Co., Ltd., Qingdao, China. DAN injection was obtained from Zhejiang Guobang Pharmaceutical Co., Ltd., China. The pure reference standard of DAN and ciprofloxacin were obtained from the Sigma-Aldrich, China. Atropine sulfate was supplied by the Shanghai Chemical Reagent Factory (Shanghai, China). Isoflurane was purchased from Hebei Yipin Pharmaceutical Co., Ltd., China and propofol injectable emulsion from Xi'an Libang Pharmaceutical Co., Ltd., China.

### Animals and implantation of an ultrafiltration probe

Six healthy males, castrated crossbred piglets (Duroc × Landrace), weighing 21–35 kg, were obtained from Harbin breed swine farm. They were housed in individual cages and allowed a 7 day acclimation period prior to the initiation of the study. Piglets had free access to water and fed antibiotic free feed twice a day.

**Figure 1:**
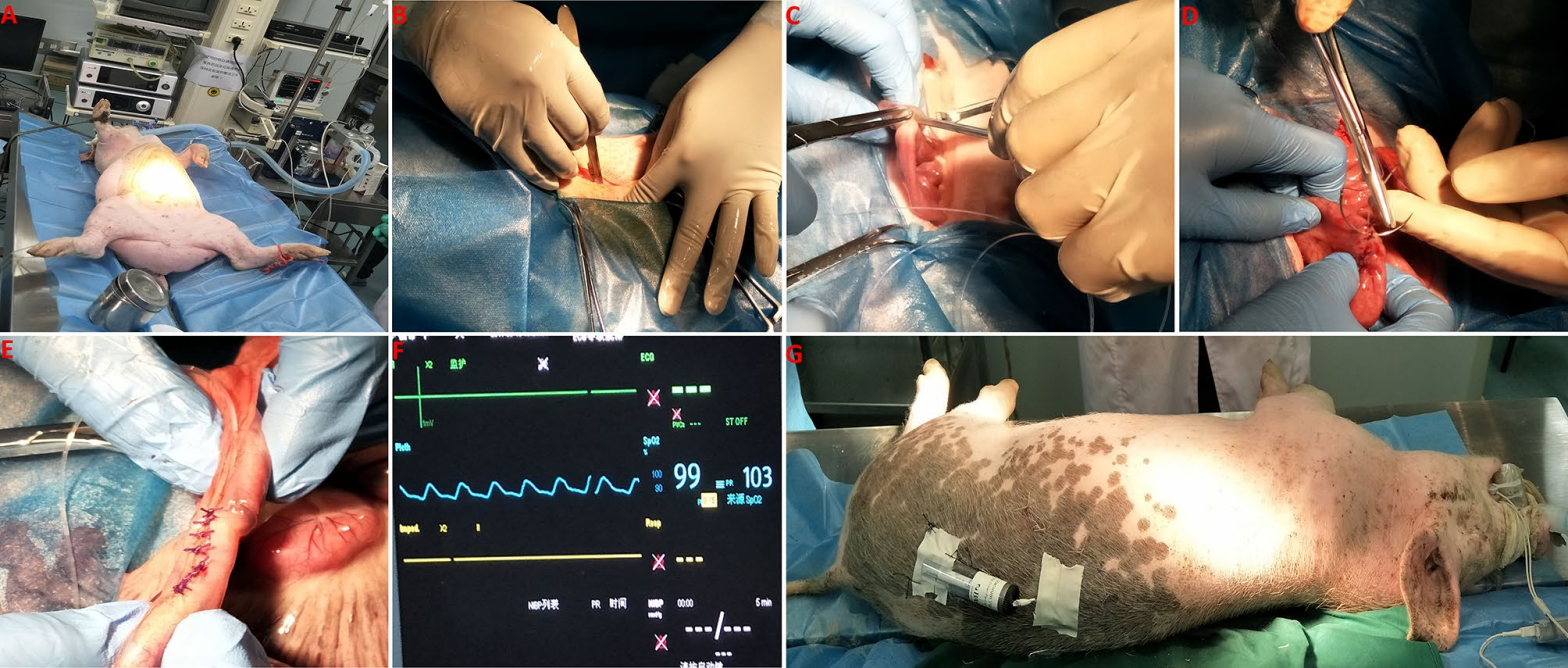
Implantation of ultrafiltration probes into the ileum of piglets. (**A**) each piglet was placed in left lateral recumbency on the surgical table and manually intubated orotracheally; (**B**) a vertical 10 cm skin incision was made approximately 10 cm above the midline of the abdomen and approximately 5 cm from the last rib; (**C**) A stab incision was made with a scalpel blade in the ileum, and an introducer needle was placed through the stab incision into the lumen of the ileum; (**D**, **E**), nodular suture of the ileum incision with 2–0 absorbable surgical suture; (**F**) the electrocardiogram, oxygen saturation, and non-invasive blood pressure via doppler were monitored throughout surgery; (**G**) the vacuum tube holder and needle are sutured to the skin.

Ultrafiltration probes were implanted into the ileum of piglets according to the previous study with modified^8^. Several BASi accessories were used for in vivo ultrafiltration sampling. These include: in vivo ultrafiltration sampling probes (MF-7023); flanged tubing connectors (MD-1510); vacuum vial needles (MD-1320) and the vacuum needle holder (MD-1322); vacutainers (MF-7024). Feed and water were withheld from the piglets for 12 h prior to surgery. Surgical procedures have been performed two days with three surgeries done each day. The piglets were pre-medicated with intramuscular administration of 0.02 mg/kg atropine sulfate prior to anesthesia. Approximately 15 min later, anesthesia was induced with 5 mg/kg propofol injectable emulsion intravenously through the ear vein. Each piglet was placed in left lateral recumbency on the surgical table and manually intubated orotracheally (Fig. 1a). General anesthesia was maintained by inhalation of 1.5–3.0% isoflurane in 100% oxygen on a circle circuit with mechanical ventilation. During surgery, the steers were given a continuous infusion of physiological saline at 10 mL/kg/h. The electrocardiogram, oxygen saturation, and non-invasive blood pressure via doppler were monitored throughout surgery (Fig. 1f).

The abdominal hair was clipped and partially disinfected with iodophor. A vertical 10 cm skin incision was made approximately 10 cm above the midline of the abdomen and approximately 5 cm from the last rib (Fig. 1b). Sharp dissection continued through the external and internal abdominal oblique muscles, transverse abdominal muscle, and peritoneum to enter the peritoneal cavity. The cecum was identified and exteriorized caudally to expose the ileocecal fold^8^. The ileocecal fold was used to identify the ileum, the location of the probe was 10 cm away from the cecum. A stab incision was made with a scalpel blade in the ileum, and an introducer needle (MR-5313, BASi, USA) was placed through the stab incision into the lumen of the ileum (Fig. 1c). The collecting end of the ultrafiltration probe was inserted through the introducer needle aborally toward the cecum so that the entire collecting apparatus was within the lumen^8^. The introducer needle was fed off the probe, and nodular suture of the ileum incision with 2–0 absorbable surgical suture obtained from Shanghai Pudong Jinhuan Medical Supplies Co., Ltd. (Fig. 1d, e). The free ends of the probes were exteriorized to the skin incision approximately 25 cm above the midline of the abdomen by using the introducer needle to create a tunnel from the abdominal cavity through the muscle layers^8^. A skin incision with a blade further exteriorized the introducer needle so that the free end of the probe could be fed through to the outside of the body. The abdominal incision was closed in three layers. The first layer included the peritoneum, transverse abdominal, and internal abdominal oblique muscles. This layer was closed in a simple continuous pattern using 2–0 absorbable surgical suture. The external abdominal oblique muscle was also closed with 2–0 absorbable surgical suture in a simple continuous pattern. Skin layer sutured with 3–0 absorbable surgical suture. The vacuum tube holder and needle are sutured to the skin (Fig. 1g). After recovery from anesthesia, they were housed in individual cages and received drug treatment to prevent infection by intramuscular injection of penicillin (1,000,000 IU/kg) and application of iodophor over the wound twice a day for 3 days.

### Sample collection and HPLC analysis

DAN was intramuscularly injected at 2.5 mg/kg body weight in each piglet after 72 h probe insertion. Blood samples (5 mL) from the brachiocephalic vein were collected into EDTA dipotassium salt tubes at 0, 0.25, 0.5, 1, 2, 4, 6, 8, 12, 24, 26, 30, and 48 h after drug administration^3^. To collect ultrafiltrate from the gastrointestinal tract, a 3 mL vacutainer (Fig. 1d) was then inserted into the needle of the vacuum vial needle holder. Approximately 300–400 µL of ultrafiltrate is collected each hour, and the probes will continuously collect ultrafiltrate. The timed interval samples were collected at 0, 1, 2, 4, 6, 8, 12, 24, 26, 30, and 48 h after drug administration by changing the vacutainer tubes. Plasma was separated by centrifugation at 3000 g for 10 min and then the supernatant was transferred into a fresh tube. All plasma and ultrafiltrate samples stored at − 80 °C till analysis.

The method for the analysis of DAN concentration was modified from that described by Schrickx and Garcia^9,10^. The HPLC system Waters 2695 was connected to a Waters 2475 fluorescence detector (λ_ex_ = 280 nm and λ_em_ = 450 nm) with a mixture of acetonitrile and aqueous solution (15:85, v/v) as the mobile phase. The aqueous solutions were prepared by dissolving potassium dihydrogenophosphate (0.020 M), phosphoric acid (0.006 M), and tetraethylammonium bromide (0.012 M) in water. The pH of the mobile phase was adjusted to 3.0 by addition of 2 N NaOH. The flow rate was set at 1.0 mL/min; A Waters C18 reverse phase column C18 (250 mm × 4.6 mm I.D.; particle size, 5 μm) was used to perform HPLC at 30 °C; and the injection volume was 10 μL.

Samples were thawed at room temperature, and 10 μL of 50 μg/mL ciprofloxacin was added to plasma (490 μL) and ileum ultrafiltrate dilution (50 μL ultrafiltrate and 440 μL mobile phase) as the internal standard. After adding 3 mL of acetonitrile, the plasma samples were shaken at 220 oscillations/min for 15 min and centrifuged at 12,000 g for 10 min. The organic layer was transferred into a fresh tube and dried at 40 °C under nitrogen stream. The residue was dissolved in the mobile phase (0.5 mL), and 10 µL injected for HPLC analysis. The ultrafiltrate sample was diluted 10 times with mobile phase and analyzed directly by HPLC without extraction after filtered.

The limit of detection (LOD) was 0.005 µg/mL and the limit of quantification (LOQ) was 0.01 µg/mL, respectively. Standard curves were linear from 0.01 to 1.5 µg/mL in plasma (R^2^ = 0.9999) and ileum ultrafiltrate (R^2^ = 0.9998). The intra-day and inter-day variation for determination in plasma ranged from 0.39 to 1.95% and 0.18 to 4.74%. The intra-day and inter-day variation for determination in ileum ultrafiltrate ranged from 0.47 to 1.42% and 0.26 to 1.86%, respectively. The recovery of DAN in plasma ranged from 88.50 to 104.80%.

### Pharmacokinetic analysis

PK parameters for DAN concentration–time data for plasma, ileum ultrafiltrate were calculated by using the WinNonlin software (version 5.2.1, Pharsight Corporation, USA). Minimum Akaike Information Criterion (AIC) was applied to discriminate the model with the best fit^11^. The data for plasma of all the six piglets were best fitted to a two-compartmental model. The data for ileum ultrafiltrate of six piglets were best described by a non-compartment model.

### Pharmacodynamics analysis

#### *Determination of MIC, MBC, and MPC for E. coli *in vitro

The MIC was determined by broth microdilution testing in accordance with the guidelines in CLSI^12^. The MBC was performed according to the guidelines in CLSI^13^. The MPC was conducted based on the previous study^14^.

### In vitro* time-kill curves*

For the in vitro time-kill curves, 9.9 mL Mueller Hinton (MH) broth with concentrations of DAN ranging from 1/2 to 32 times of the MICs (0.25–16 µg/mL) for *E. coli* HP501 (O_158_, MIC = 0.5 µg/mL) and 0.1 mL bacteria (0.5 Mc Farland standards, approximately 10^8^ CFU/mL) were co-incubated at 37 °C, which were tested separately. The inoculum sizes of the organisms used to generate the time-kill curves were approximately 10^6^ CFU/mL. An aliquot of 100 µL from each tube was placed in 0.9 mL of MH broth (1/10 dilution, 10^–1^). Then, 100 µL of MH broth were serially diluted in 0.9 mL to give dilutions of 10^–2^, 10^–3^, 10^–4^, 10^–5^, 10^–6^, 10^–7^, 10^–8^, 10^–9^ and 10^–10^. Five 20 µL drops of each dilution were dropped onto MH broth plates for determination of the CFU at 0, 1, 2, 4, 6, 8, 12 and 24 h. Finally, bacteria were counted after the plates were incubated at 37 °C for 24 h.

### PK-PD integration for DAN in ileum ultrafiltrate

After i.m. dosing of DAN, the ex vivo AUC at 24 h (AUC_24h_)/MIC ratio and C/MIC were determined for ileum ultrafiltrate by using in vitro MIC data and in vivo PK parameters. The relationship between the AUC_24h_/MIC, C_max_/MIC and the log_10_ difference between the initial bacterial count (CFU/mL) and the bacterial count after 24 h of incubation was established for ultrafiltrate by using the inhibitory effect sigmoid E_max_ equation ^15^. This model is described by the following equation:

$$E={E}_{max}-\frac{\left({E}_{max}-{E}_{0}\right)\cdot {C}^{N}}{{EC}_{50}^{N}+{C}^{N}},$$

where E is the antibacterial effect determined as the change in the bacterial count (log_10_ CFU/mL) in the ultrafiltrate sample after 24 h of incubation compared to the initial log_10_ CFU/mL; E_0_ and E_max_ are the changes in log_10_ difference in bacterial count between 0 and 24 h in the control sample and in the DAN containing samples, respectively; EC_50_ is the AUC_24h_/MIC or C/MIC producing 50% of the maximal antibacterial effect; C is the AUC_24h_/MIC or C/MIC in the effect compartment; N is the Hill coefficient, which describes the steepness of the (AUC_24h_/MIC)-effect curve or C/MIC-effect curve. DAN has inhibitory effect on bacterial growth. The E_max_ and E_0_ were obtained from the ex vivo PD test. These PD parameters EC_50_, C, N were calculated by using the WinNonlin software (version 5.2.1, Pharsight Corporation, USA).

The values of PK/PD parameters AUC_24h_/MIC and C_max_/MIC required to achieving the three levels of antibacterial effect of DAN in ileum ultrafiltrate: (1) E = 0, bacteriostatic action (no change in bacterial count after 24 h of incubation); (2) E = -1, bactericidal action (a 99.9% decrease in the bacterial count); (3) E = − 3, bacterial elimination (the lowest AUC_24_/MIC that produced a reduction in the count to 10 CFU/mL).

### Calculation of the administered dosage

The dose was calculated by the using the following formula^15^:

$$\mathrm{Dose}=\frac{\left(AUC/MIC\right)\times CL}{fu\times F}\times MIC,$$

where AUC/MIC is the targeted endpoint for optimal efficacy; CL is clearance per day; MIC is minimum inhibitory concentration ; fu is free fraction of drug in ileum ultrafiltrate (from 0 to 1); F is the bioavailability factor (from 0 to 1). In this study, fu is 1 because the ileum ultrafiltrate collected by the ultrafiltration probe contained only the free state of DAN.

The dose was calculated respectively by the formula above, based on the PK/PD parameters AUC_24h_/MIC and C_max_/MIC required to achieve the bacteriostatic action, bactericidal action and bacterial elimination.

### Statistical analyses

Statistical analysis was undertaken by analysis of variance using GraphPad Software Prism 6 (version 6.01). Differences between the means of the plasma group and ileum ultrafiltrate group were assessed using two-tailed paired Student’s t test. All data were presented as the mean ± SD, where P < 0.05 was considered a statistically significant difference.

## Results

### Pathogenicity test

The results of *E. coli* virulence test for mice were presented in Table 1. Mice in each group showed different degrees of death when different concentrations of *E. coli* were inoculated into the peritoneal cavity of mice. The strain HP501 among them had the strongest lethal ability to mice. Therefore, an EPEC *E. coli* strain HP501 (O_158_) was selected for subsequent PD experiments.

**Table 1:** The results of *E. coli* virulence test for mice.

Strain	Serotype	MIC (µg/mL)	Mortality no./Total no.			
			10^7^ CFU/mL	10^8^ CFU/mL	10^9^ CFU/mL	Blank
HP65	O_158_	0.5	1/3	2/3	2/3	–
HP189	O_158_	0.5	0/3	0/3	1/3	–
HP232	O_158_	0.5	0/3	1/3	1/3	–
HP501	O_158_	0.5	2/3	3/3	3/3	–
Sterile broth	–	–	–	–	–	0/3

### Pharmacokinetics analysis

**Figure 2:**
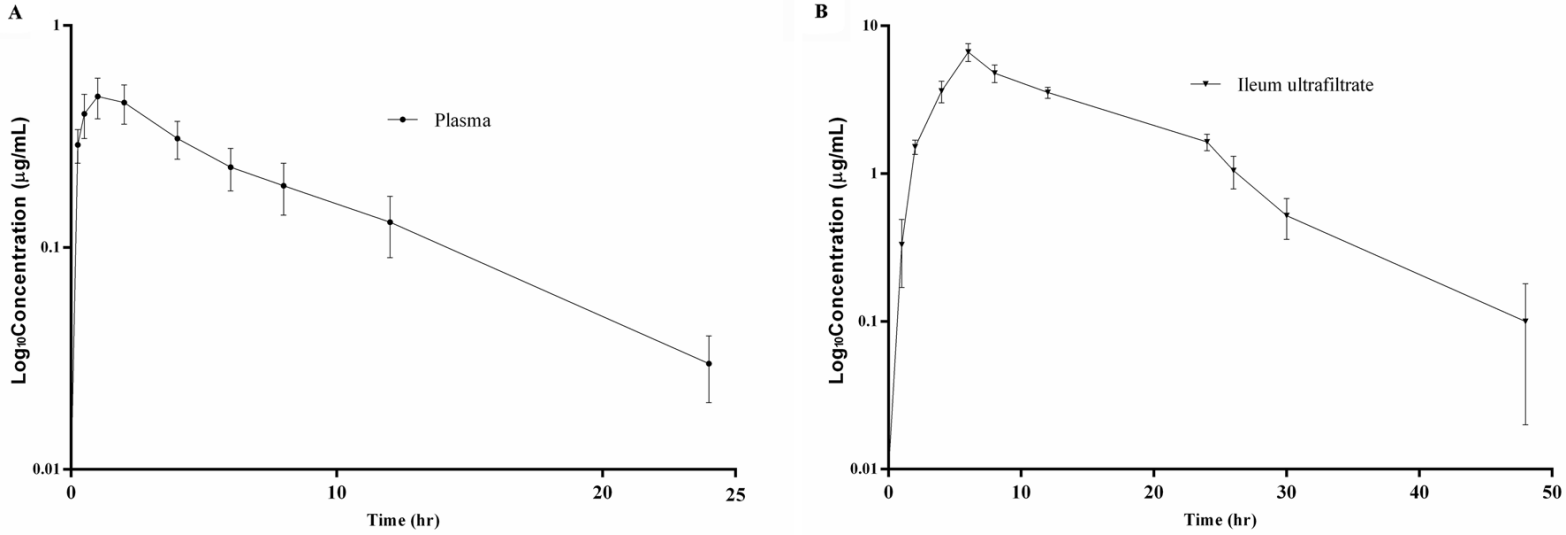
Semi-logarithmic plot of concentration–time profile of plasma at 0, 0.25, 0.5, 1, 2, 4, 6, 8, 12 and 24 h (**A**) and ileum ultrafiltrate at 0, 1, 2, 4, 6, 8, 12, 24, 26, 30 and 48 h (**B**) after DAN i.m. administration at a dose regimen of 2.5 mg/kg in piglets. Values are means ± SD (n = 6).

Semi-logarithmic plot of concentration–time profile of plasma at 0, 0.25, 0.5, 1, 2, 4, 6, 8, 12 and 24 h (A) and ileum ultrafiltrate at 0, 1, 2, 4, 6, 8, 12, 24, 26, 30 and 48 h (B) after DAN i.m. administration at a dose regimen of 2.5 mg/kg in piglets are presented in Fig. 2. In Table 2, the DAN concentration in plasma reached its maximum at 1.10 ± 0.03 h, but it was 6.00 ± 0.00 h in the ileum ultrafiltrate. The mean C_max_ of the ileum is 13.59 times that of plasma. The elimination half-life (T_1/2_) in the ileum ultrafiltrate (6.84 ± 1.49 h) was shorter than those in plasma (7.58 ± 3.20 h).

**Table 2:** PK parameters for DAN in plasma and ultrafiltrate after i.m. administration at a dose of 2.5 mg/kg (*n* = 6).

Parameter	Unit	Plasma	Ultrafiltrate
T_1/2_	h	7.58 ± 3.20	6.84 ± 1.49
AUC_0–24_	h*µg/mL	4.31 ± 0.88	85.32 ± 8.57**
T_max_	h	1.10 ± 0.03	6.00 ± 0.00**
C_max_	µg/mL	0.49 ± 0.11	6.66 ± 0.92**
CL	L/h/kg	15.94 ± 5.53	29.56 ± 3.06**
V_ss_	L/kg	5.14 ± 0.74	0.29 ± 0.061**
MRT	h	NA	14.16 ± 1.52

T_1/2_: elimination half-life of the drug; AUC_0–24_: area under the curve of plasma or ultrafiltrate concentration–time; T_max_: the time point of maximum plasma concentration of the drug; C_max_: the maximum plasma concentration; CL: body clearance; V_ss_: volume of distribution at steady state; MRT: mean residence time; NA: not applicable.**P ˂ 0.01 for comparison of plasma and ultrafiltrate.

### Pharmacodynamics analysis

#### *MIC, MBC, and MPC against E. coli *in vitro

The MIC of DAN in MH broth against *E. coli* (O_158_) was 0.5 µg/mL and its corresponding MBC values was 0.5 µg/mL. The MPC of DAN in MH broth was 4 µg/mL (Table 3).

**Table 3:** MIC, MBC, and MPC (µg/mL) of DAN against *E. coli* HP501 (O_158_).

Matrix	MIC (µg/mL)	MBC (µg/mL)	MPC (µg/mL)
MH broth	0.5	0.5	4

### In vitro* time-kill curves*

**Figure 3:**
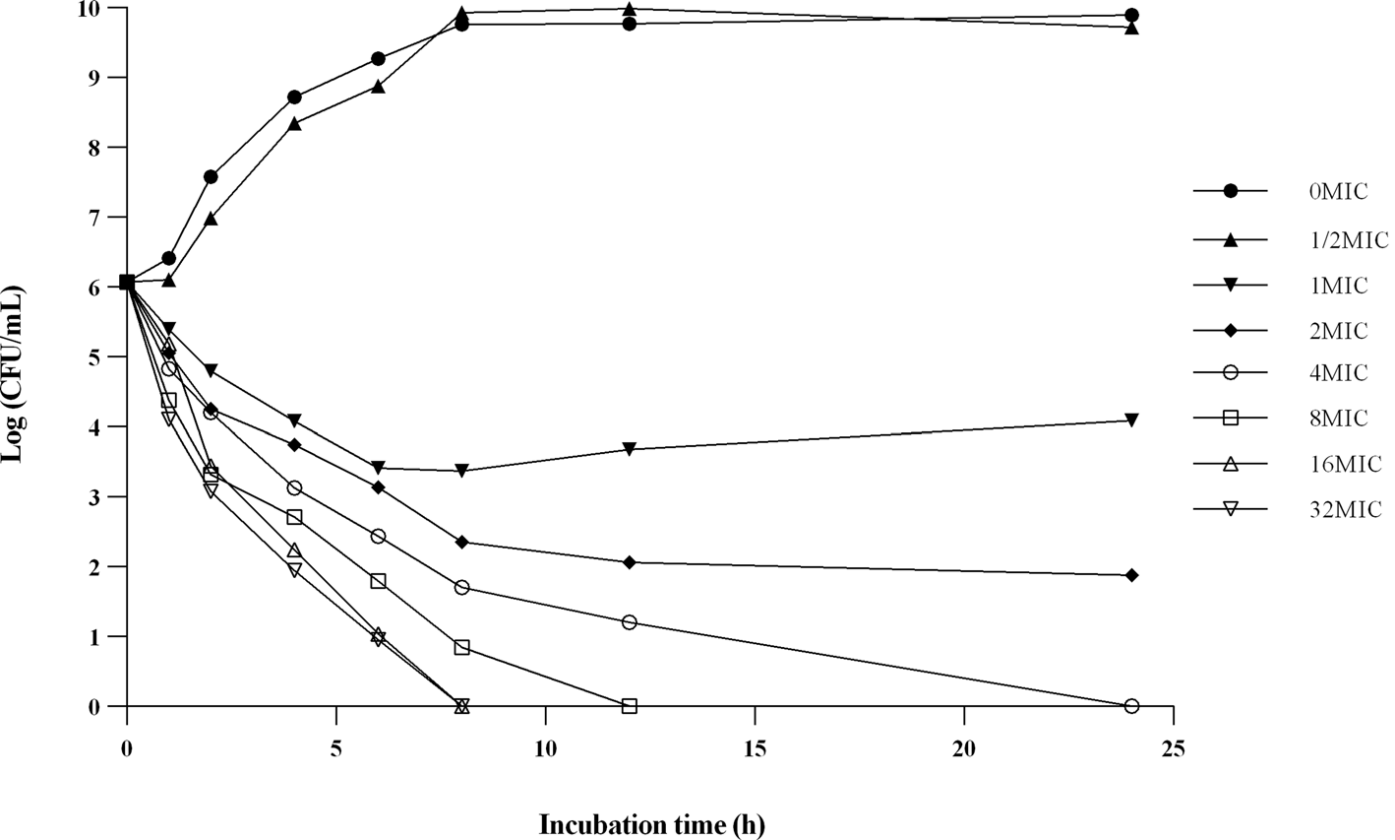
The in vitro time killing curve of DAN against *E. coli* HP501 (O_158_, MIC = 0.5 μg/mL).

The in vitro time-kill curves were showed in Fig. 3. The bactericidal activity increased with increasing concentration of DAN in MH broth medium. The bacteria were all killed after 8 h of co-cultured with MH broth which containing DAN concentration was 16 times or 32 times the MIC. This result indicates that the killing mechanism of DAN against *E. coli* is concentration-dependent.

### PK-PD integration for DAN in ileum ultrafiltrate

The PK-PD integration parameters for the in vivo PK data and the MICs determined in vitro for ileum ultrafiltrate are presented in Table 4. The mean AUC/MIC and AUC/MPC were 170.64 ± 17.14 h and 21.33 ± 2.14 h for *E. coli* HP501 (O_158_) in ileum ultrafiltrate after i.m. administration of DAN at 2.5 mg/kg. The time for which the concentration in ultrafiltrate remained above the MIC and MBC were 29.76 ± 0.68 h and 19.32 ± 0.19 h, respectively. The mean C_max_/MIC ratio and C_max_/MPC were 13.32 ± 1.84 and 1.67 ± 0.23, respectively.

**Table 4:** PK/PD integration parameters for DAN in ileum ultrafiltrate after i.m. administration at a dose of 2.5 mg/kg. (n = 6).

Parameter	Units	Mean ± SD
AUC/MIC	h	170.64 ± 17.14
AUC/MPC	h	21.33 ± 2.14
C_max_/MIC	–	13.32 ± 1.84
C_max_/MPC	–	1.67 ± 0.23
T > MIC	h	29.76 ± 0.68
T > MBC	h	19.32 ± 0.19

**Figure 4:**
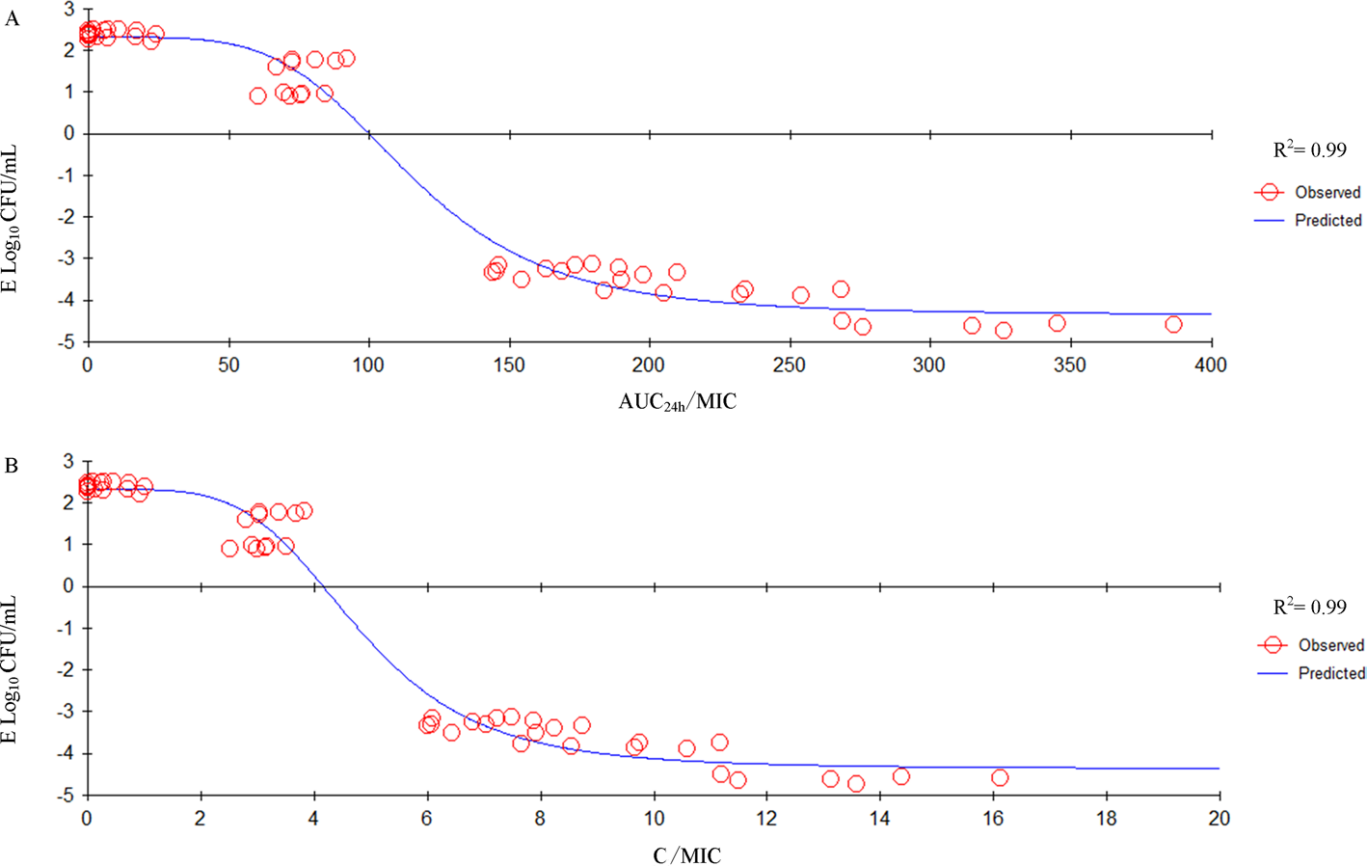
A, B: Plots of antibacterial effect [E, log_10_ (CFU/mL)] versus respectively AUC_24h_/MIC and C_max_/MIC of DAN in ileum ultrafiltrate in the ex vivo PK/PD model against *E. coli* with an inoculum size of 1.49 × 10^6^ CFU/mL, based on inhibitory effect sigmoid E_max_ model. Where, R^2^ is the correlation coefficient; MIC: minimum inhibitory concentration; AUC_24h_: area under the concentration–time curve from 0 to 24 h after DAN administration; C_max_: the maximum concentration during the dosage interval. The circles represent the values observed for individual animals, and the curve is the line of best fit.

AUC_24h_/MIC and C/MIC values were generated according to the PK parameters in vivo in ultrafiltrate and the MIC in MH broth. The relationship between ex vivo antimicrobial efficacy and PK/PD surrogate markers (AUC_24h_/MIC or C_max_/MIC) is presented in Fig. 4 by the inhibitory effect sigmoid E_max_ equation. Both the AUC_24h_/MIC and C/MIC had a good correlation with antimicrobial efficacy (R^2^ = 0.99). The estimated E_max_ (Log_10_ CFU/mL), E_0_ (Log_10_ CFU/mL), EC_50_ (h), and N (slope) are showed in Table 5. According to the inhibitory effect sigmoid E_max_ equation:$E=2.32-\frac{6.67\cdot {C}^{4.51}}{{114.78}^{4.51}+{C}^{4.51}}$, the calculated mean AUC_24h_/MIC, is the C in the inhibitory effect sigmoid E_max_ equation, for ileum ultrafiltrate that produced bacteriostasis, bactericidal activity, and elimination of bacteria were 99.85, 155.57, and 218.02 h, respectively (Table 5); the calculated mean C_max_/MIC for ileum ultrafiltrate that produced bacteriostasis, bactericidal activity, and elimination of bacteria were 4.14, 6.49, and 9.05, respectively (Table 5).

**Table 5:** The values of PK/PD parameters AUC_24h_/MIC and C_max_/MIC required to achieving various levels of antibacterial effect of DAN.

Parameter	Unit	PK/PD fitting parameters	
		AUC_24h_/MIC	C_max_/MIC
E_max_	Log_10_ CFU/mL	2.32 ± 0.10	2.32 ± 0.10
E_0_	Log_10_ CFU/mL	− 4.36 ± 0.17	− 4.37 ± 0.17
EC_50_	h	114.78 ± 3.79	4.79 ± 0.16
N	–	4.51 ± 0.40	4.46 ± 0.40
AIC	–	131.23	131.25
R^2^	–	0.99	0.99
E_max_–E_0_	Log_10_ CFU/mL	6.67 ± 0.20	6.69 ± 0.20
E = 0 (Bacteriostatic)		99.85	4.16
E = − 3 (Bactericidal)		155.57	6.49
E = − 4 (Elimination)		218.02	9.05

E_0_ and E_max_ are the changes in log_10_ difference in bacterial count between 0 and 24 h in the control sample and in the DAN containing samples, respectively; EC_50_ is the AUC_24h_/MIC or C_max_/MIC producing 50% of the maximal antibacterial effect; C is the AUC_24h_/MIC or C_max_/MIC in the effect compartment; N is the Hill coefficient, which describes the steepness of the (AUC_24h_/MIC)-effect curve or C_max_/MIC-effect curve.

### Calculation of the administered dosage

Three different dosages (1.49 mg/kg, 2.42 mg/kg, and 3.24 mg/kg) were calculated respectively based on AUC_24h_/MIC ratio for bacteriostatic, bactericidal and bacterial eradication in Table 6.

**Table 6:** Different dosages were calculated respectively based on AUC_24h_/MIC ratio for bacteriostatic, bactericidal and bacterial eradication.

E (Log_10_ CFU/mL)	Dosage (mg/kg)
E = 0 (bacteriostatic)	1.49
E = − 3 (bactericidal)	2.42
E = − 4 (elimination)	3.24

## Discussion

DAN is a fluoroquinolones antibacterial that has wide spectrum of antimicrobial activity against most gram-negative bacteria and some gram-positive bacteria^3^. Improper use of antibiotics that inappropriate dosing, dosing interval, dosing time, dosing route, dosing conditions lead to poor treatment effect and serious bacterial resistance^16^. To date, PK-PD modeling has been widely used to improve dosing regimens of approved drugs and individualized treatment^17^. Therefore, it was investigated that the PK, PD, and PK-PD integration of DAN in piglet model against *E. coli* in this study.

PK data of DAN in plasma has been described in several species, including cattle, cows, ewes, sheep, goat, camel, brown trout, rabbits, chicken, turkeys, ducks, donkeys, horses and pigs^3^. After i.m. administration of DAN at a dose of 2.5 mg/kg body weight in piglets, the PK parameters including that the time to reach to maximum concentration (T_max_) in plasma, the peak drug concentration (C_max_), the area under the curve (AUC_0-t_), and the elimination half-life (T_1/2β_) do not differ to a large extent from previous studies^18–20^.

Previous publications were mainly focused on PK data of DAN in plasma but ignored the concentrations of antimicrobial agents in their target sites. Therefore, the PK data in both plasma and ileum were analyzed in our study. Luminal gastrointestinal tract (GIT) fluid could be collected by a wide variety of approaches. For instance, euthanasia of multiple animals to collect GIT contents at multiple time points, requiring a large number of trial animals and yielding a relatively low number of animals per time point^21^. Several cannulas and tubes have been used to collect GIT fluid from ileum, rumen and duodenal cannulas in animals^15,22^. These cannulas must be anchored to the abdominal wall which results in disrupting normal intestinal motility, carrying inherent risks of leakage, dislodgement, and peritonitis and significant welfare concerns. In addition, antimicrobial drugs such as fluoroquinolones can be extensively bound to the intestinal contents, but intestinal concentrations should take into account only the unbound and active drug for subsequent PD studies. Ultrafiltration probe technology can solve this problem well in consideration of animal welfare. Ultrafiltration probes have been used to collect interstitial fluid (ISF) in a number of species^8,23,24^. In this study, ultrafiltration sampling probes were successfully placed in the ileum of piglets with the aid of anesthetic, and ileum ultrafiltrate contained only free drugs was collected at different time points. These free drug concentrations could finally give a value of an exposure parameter for the digestive tract (AUC_ileum_) that, when combined with the relevant MIC in ileum ultrafiltrate, would predict the efficacy of the drug dosing regimen on the intestinal bacterial population.

A study described the PK variables of DAN in gastrointestinal content from ileum, jejunum, caecum and colon in healthy and in *salmonella typhimurium* infected pigs^25^. Lindecrona et al. administered DAN intravenously and found maximum concentration (4.46 µg/mL) at 2 h. While, in our study, piglets were administered intramuscularly and C_max_ value (6.66 ± 0.92 µg/mL) was observed at 6 h. The difference in time to reach the C_max_ value of DAN may be due the different administration routes. The AUC_ileum_/AUC_plasma_ ratio and MRT were 19.89 and 14.16 ± 1.52 h, higher than 10.97 and 9.86 h as reported by Lindecrona et al.. DAN concentrations in the gastrointestinal tract is much higher than the corresponding plasma concentrations in both the study in ours and Lindecrona et al.’ s, indicating that intestinal drug concentration could better predict the intestinal exposure of DAN than the plasma concentration.

There are no previous studies reported that the AUC/MPC for DAN against *E. coli*. In this study, the AUC/MPC ratio is 21.33 ± 2.14 (Table 4), which similar to the previous study (18.8 h) performed enrofloxacin against *E. coli*^15^. Previous study illustrated that ciprofloxacin treatment at AUC/MPC ratios of 22 could prevent the resistant mutants of *E. coli* with an inoculum sizes of 10^10^ CFU/mL^26^. The C_max_/MPC ratio (1.67 ± 0.23) obtained in our study was close to that reported in previous study (1.4) for enrofloxacin against *E. coli*.

Rational use of antibiotics requires extensive knowledge of the infectious bacteria and the PK and PD of the drugs used^27^. The efficacy of antimicrobial agents against bacterial pathogens is classically predicted from PK/PD studies. In general, to be efficacious against the pathogens responsible for an infection, drug dosing regimens should ensure that the optimal value of the PK/PD index correlated with the antibacterial activity of the drug is achieved^28^. For fluoroquinolones, a C_max_/MIC ≥ 10 or an AUC_24h_/MIC ≥ 125 is the PK/PD index that reach antimicrobial efficacy^3,29,30^. This finding was also true in this study (Table 5), both in vitro kill curves indicated that the higher the concentration of DAN, the better the bactericidal effect. Moreover, both the AUC_24h_/MIC and C_max_/MIC had a good correlation with antimicrobial efficacy (R^2^ = 0.99) by the inhibitory effect sigmoid E_max_ equation. The calculated mean AUC_24h_/MIC for ileum ultrafiltrate that achieved bacteriostatic, bactericidal, and eradication were 99.85, 155.57, and 218.02 h. These results are consistent with other studies which AUC_24h_/MIC ratios for the quinolones should be at least 125 for optimal bactericidal efficacy against gram-negative pathogens^31^.

Previous studies have found that the increase in antibiotic resistance in the intestinal flora was mainly due to the abuse of antimicrobials in unsuitable routes of administration^32,33^. Therefore, three different doses (1.49 mg/kg, 2.42 mg/kg and 3.24 mg/kg) were calculated respectively based on the PK/PD index (AUC_24h_/MIC ratio) for bacteriostatic, bactericidal and bacterial eradication in the present study. However, it is necessary to design experiments with either naturally diseased animals or disease models to establish the lowest AUC/MICs that lead to the bacterial elimination in vivo^3^. Such experiments will further provide a more rational basis for the selection of optimal dosage schedules for DAN.

## Conclusion

In this study, ultrafiltration probes were successfully placed in the ileum of piglets to collect ileum ultrafiltrate. The mean PK/PD index (AUC_24h_/MIC) for ileum ultrafiltrate that achieved bacteriostatic, bactericidal, and eradication were 99.85, 155.57, and 218.02 h, respectively. Three different dosages (1.49 mg/kg, 2.42 mg/kg, and 3.24 mg/kg) were calculated respectively based on AUC_24h_/MIC ratio above, which might provide a novel approach to the rational design of dosage schedules.
